# Enhanced Interface Structure and Properties of Titanium Carbonitride-Based Cermets with the Extra Solid Phase Reaction

**DOI:** 10.3390/ma10091090

**Published:** 2017-09-15

**Authors:** Nan Lin, Yuehui He, Xiyue Kang

**Affiliations:** 1 College of Materials Science and Engineering, Hunan University, Changsha 410082, China; 2 State Key Laboratory for Powder Metallurgy, Central South University, Changsha 410083, China; xiyuekang@csu.edu.cn

**Keywords:** cermets, transmission electron microscopy, interface structure, mechanical properties, abrasion resistance

## Abstract

In this paper, the influence of the extra solid phase reaction on the interface structure and mechanical properties of titanium carbonitride-based cermets were investigated. The extra solid phase reaction in the preparation process of cermets could induce the formation of a core/rim/binder interface with the coherent structure and reinforce the interface bonding strength in cermets. The existence of a coherent structure interface can inhibit crack spread and improve the toughness and abrasion resistance of titanium carbonitride-based cermets significantly. Cermets can exhibit the high hardness Rockwell Hardness A (HRA) 92.3, fracture toughness of 11.6 MPa·m^1/2^, and transverse rupture strength of 2810 MPa.

## 1. Introduction

Owing to high hardness, perfect abrasive resistance, excellent oxidation resistance, and low friction coefficient to steel and cast iron, Ti(C,N)-based cermets are widely used in the high-speed cutting of metallic materials, which are composed of Ti(C,N) ceramic particles and a Co/Ni binder phase [1,2,3,4]. The inferior toughness of Ti(C,N) cermets, which is caused by the poor wettability between Ti(C,N) particles and the Co/Ni binder phase, can limit the application field of Ti(C,N) cermets [5,6]. The addition of carbides can induce the formation of a rim phase between Ti(C,N) particles and the Co/Ni binder phase, which can improve the wettability and strengthen the bonding between the Ti(C,N) and the Co/Ni binder phase. However, during the conventional vacuum liquid-phase sintering process, the rim phase can cause internal stress due to the mismatch between core and rim lattices, which may bring about a sharp decreasing of mechanical properties in Ti(C,N) cermets, cause the breakage of cermets tools in cutting processes, and damage the lifetime of Ti(C,N)-based cermet tools inevitably [7,8,9].

The mechanical properties of Ti(C,N)-based cermets are influenced by the sintering densification process, which can determine the interface structure of the core/rim/binder in ceramics. For Ti(C,N)-based cermets, the detailed studies of crystallographic characterizations for core/rim grain boundaries show that the interface structure of core/rim grain boundaries is an incoherent interface [10]. However, a coherent core/rim interface can be found in (Ti,Ta)(C,N)-based cermets developed by a mechanically induced, self-sustaining reaction [11]. However, there are few studies on the effect mechanism among the sintering process, interface structure feature, and mechanical properties of Ti(C,N)-based cermets. Herein, we report an innovative method combining a solid phase reaction and a liquid phase sinter-hot isostatic pressing (HIP) to prepare Ti(C,N)-based cermets with a coherent interface in core/rim/binder phase grain boundaries, and illuminate a reasonable mechanism among interface structure, mechanical properties, and abrasion resistance.

## 2. Experimental Procedures

The Ti(C_0.5_,N_0.5_)-20 wt %WC-8 wt %Mo_2_C-4 wt %TaC-9 wt %Ni-9 wt %Co cermets were prepared in the present work. Ti(C_0.5_,N_0.5_) (2.5 μm), WC (1.2 µm), Mo_2_C (1.5 µm), TaC (1.8 μm), Co (0.8 μm), and Ni (1.2 μm) powders were used as raw materials, which were milled by a planetary high-energy ball-mill (XQM-2, Kexi, Nanjing, China) in methanol for 48 hours at the milling speed of 250 r/min and with the ball-to-powder weight ratio of 6:1. Subsequently, the powders were mixed with 3 wt % paraffin, granulated, and compressed into a rectangular plate under the pressure of 200 MPa. The sintering was carried out in a vacuum furnace with 1 × 10^−2^ Pa and then heated up to 1300 °C for 120 min to conduct the extra solid phase reaction. Then, sintering was conducted by a sinter-HIP at 1510 °C for 60 min in argon with an air-pressure of 5 MPa. Cermets with dimensions of 20 × 6.5 × 5.25 mm^3^ were prepared for microstructural analysis and mechanical properties measurement.

The microstructure observation of densified cermets was performed by an FEI NanoSEM230 scanning electron microscope (SEM) (Thermo Fisher Scientific, Hillsboro, OR, USA). Transmission electron microscopy (TEM) observations were conducted in JEM-2100-200 kv (JEOL, Tokyo, Japan). Hardness was tested by a Rockwell hardness tester (INSTRON, Boston, MA, USA) under a constant load of 60 kg as well as a Vickers hardness tester (Huayin Testing Instrument Co., Ltd., Laizhou, China) under a constant load of 30 kg. The transverse rupture strength was measured at room temperature with a loading speed of 2 mm/min by the three-point bending technique with INSTRON 3369 (INSTRON, Boston, MA, USA). Five specimens were tested for transverse rupture strength. The fracture toughness was determined by measuring the length of cracks in the Vickers indentation and calculations with the Shetty formula [12]. The abrasion resistance of cermets was tested in a high-speed reciprocating friction testing machine (HRS-2M, Zhongkekaihua, Lanzhou, China) for 10 min at the applied load of 50 N by sliding against a WC-6Co ball with a rotating speed of 300 r/min.

## 3. Result and Discussion

**Figure 1:**
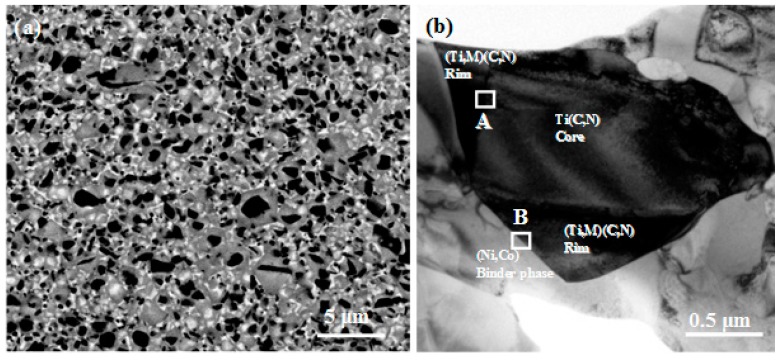
SEM micrographs (**a**) and TEM observation (**b**) of the microstructure in Ti(C,N)-based cermets prepared by solid phase reaction and liquid phase sinter-HIP.

The microstructures of Ti(C,N)-based cermets, which were synthesized by the extra solid phase reaction and sinter-HIP, are shown in Figure 1. Figure 1a shows a typical core-rim-binder microstructure in Ti(C,N)-based cermets. The black core phases are Ti(C,N) particles, which were not dissolved and reacted in the sintering process. Moreover, the gray rim phases are the solid solutions, which are composed of (Ti,W,Mo,Ta)(C,N) [13,14]. The white binder phase is observed along the rim phase, which can bring about a larger mean free path and improve the mechanical properties.

The measured properties of Ti(C,N)-based cermets are listed in Table 1, together with comparisons from the literature. The hardness, transverse rupture strength, and fracture toughness of cermets prepared by the solid phase reaction and liquid phase sinter-HIP were measured as 92.3 HRA, 2810 MPa, and 11.6 MPa·m^1/2^, respectively (see more transverse rupture strength details in Figure S1 in supplementary data). The relative densities of cermets prepared by direct liquid phase sinter-HIP, and by solid phase reaction and liquid phase sinter-HIP were 99.6% and 99.8%, which means that the extra solid phase reaction can enhance the densification process, reduce the porosity, and improve the transverse rupture strength of cermets. Compared with the values of mechanical properties in Ti(C,N)-based cermets manufactured by the sinter-HIP, vacuum sintering, and hot pressing [10,15,16], the present Ti(C,N)-based cermets exhibited an excellent mechanical properties. Therefore, the extra solid phase reaction can improve the mechanical properties of Ti(C,N)-based cermets, especially the transverse rupture strength.

**Figure 2:**
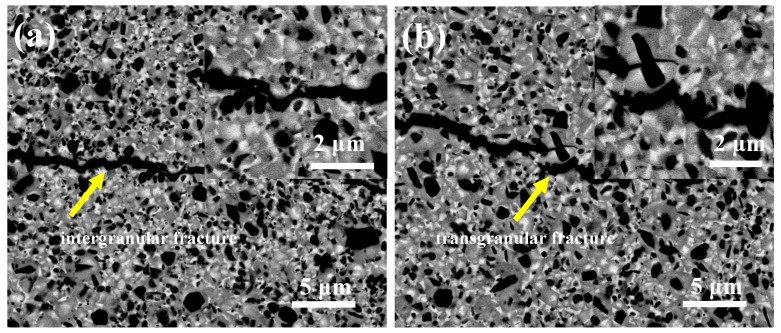
SEM micrographs of crack with Ti(C,N)-based cermets prepared by direct liquid phase sinter-HIP (**a**), and solid phase reaction and liquid phase sinter-HIP (**b**).

Figure 2 shows the crack propagation of Ti(C,N)-based cermets. The indentation crack is extended along the grain boundary in cermet prepared by direct liquid phase sinter-HIP, and the Ti(C,N) grains cannot influence the propagation of crack path distinctly, as shown in Figure 2a. Figure 2b shows that the mode of crack deflection in cermet prepared by the solid phase reaction and sinter-HIP changed into a trans-granular fracture. This hints that the excellent bond strength of the core/rim/binder grain boundary in Ti(C,N)-based cermets may influence the crack spread in the crack extension direction, generate an amount of resistance to the crack propagation, and enhance the toughness of cermets drastically.

**Figure 3:**
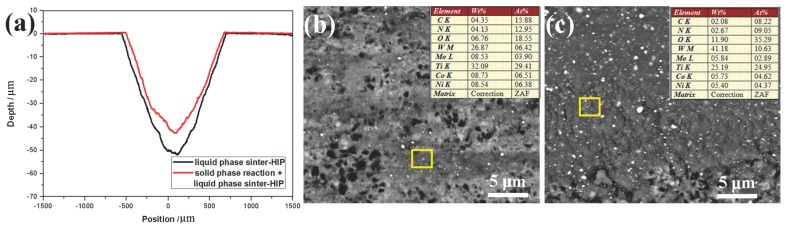
The wear depth of cermets (**a**) and SEM images of the wear track for cermets with the direct liquid phase sinter-HIP (**b**) and extra solid phase reaction (**c**).

The wear properties and compositions of abrasive dust of Ti(C,N)-based cermets with the different sintering processes are shown in Figure 3. Figure 3a shows that the extra solid phase reaction can contribute to the reduction of the depth value of the wear track and wear volume in the process of abrasion. It can be seen from Figure 3b,c that the W element contents of the abrasive dust are 26.87 wt % and 41.18 wt % in the Ti(C,N)-based cermets with the extra solid phase reaction and direct liquid phase sinter-HIP. This means that Ti(C,N)-based cermets with the extra solid phase reaction can possess higher abrasion resistance and achieve a larger quality of W element from the WC-Co ball in the wear track of cermets. According to Table 1 and Figure 2, the solid phase reaction and sinter-HIP can bring about the higher transverse rupture strength and fracture toughness of cermets, which can induce the outstanding bond strength of the interface among grain boundaries and improve the abrasion resistance.

**Figure 4:**
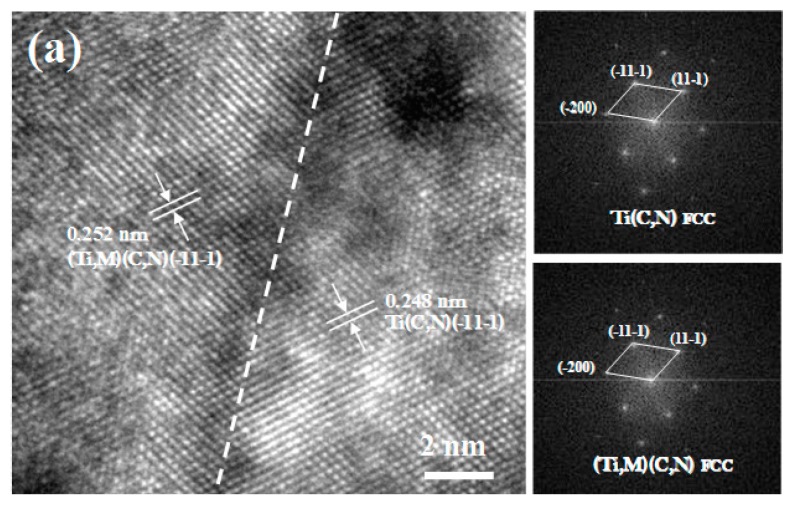

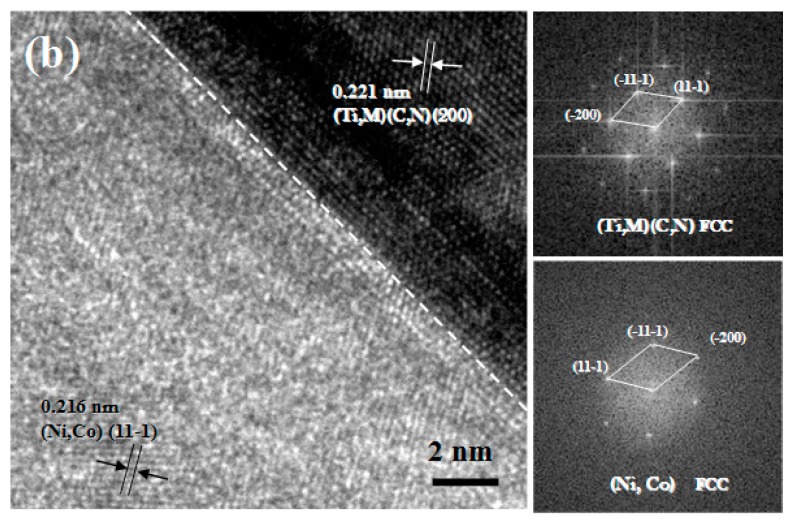
HRTEM micrograph of the core/rim interface (**a**) (corresponding to region A in Figure 1) and the rim/binder phase interface in prepared cermets (**b**) (corresponding to region B in Figure 1).

To investigate the morphology and structure of the cermets in depth, HRTEM of cermets were performed. Figure 4 shows the HRTEM micrograph of the core/rim and rim/binder grain boundaries, and the Fast Fourier Transformation patterns are shown in the insets. It can be seen from Figure 4a that the core and rim grain of prepared cermets match well at the boundary. The core and rim crystals have no spatial orientation difference or obvious lattice distortion. The measured interplanar distances for the (−11−1)_core_ and (−11−1)_rim_ are 0.248 nm and 0.252 nm, respectively, which reveals the high coherence of the interface (the lattice mismatch is 0.98%). The excellent matching of neighbor crystal lattices at the core/rim grain boundary increases the core/rim interface stability and improves the bond strength of the grain boundary. Figure 4b shows that there is a excellent match between rim and binder phases at the interface. The rim grain and binder phase have an orientation relationship of (200)_rim_//(11−1)_binder_ at the interface, whose interplanar distances are 0.221 nm and 0.216 nm, respectively. There is the misfit of 2.3% between rim and binder phase at the interface. The lattice distortion between the rim/binder phase boundary reduces the misfit of rim and binder crystals along the (200)_rim_ and (11−1)_binder_ plane and enhances the bond strength of the rim/binder grain boundary.

In the sintering process of Ti(C,N)-based cermets, Ti(C,N) particles can dissolve in the binder phase at high temperatures, and Ti, C, and N elements could reprecipitate as Ti(C,N) from the binder phase. The addition of carbides can precipitate as the rim phases in the form of a complicated (Ti,M)(C,N) solution. During the direct liquid phase sintering process, the fast heating may cause an unordered formation of the microstructure, induce the generation of core/rim/binder interfaces with incoherent structure, and form a Ti(C,N)/binder grain boundary with weak bond strength, which can cause a decrease in the toughness [10,17]. Compared to the conventional direct liquid phase sintering method for controlling the formation of core/rim structure in cermets [9,10], the solid phase reaction and liquid phase sinter-HIP method can solve the problem of a lack of bond strength at grain boundaries. During the sintering process, the formation of the rim structure can be achieved at the solid stage sintering. Mo_2_C and TaC can react with Ti(C,N) particles and form the rim phase during the solid stage sintering before 1300 °C. The solid stage sintering can enhance the diffusion reaction between WC and Ti(C,N) particles, and form the rim phase (see Figures S2 and S3 in supplementary data). Moreover, the rim phase can grow along the crystal structure of Ti(C,N) core by an oriented attachment mechanism, and achieve the structural consistency of the core/rim interface with various constituents. Moreover, in the liquid phase sintering process, the dissolution of (Ti,M)(C,N) from the Ti(C,N)/(Ti,M)(C,N) core/rim grains can generate a (Ti,M)(C,N)-rich layer at the interface between (Ti,M)(C,N) and the binder phase. The difference in the core/rim/binder interface relationships of cermets fabricated by diverse sintering methods can cause variation in the toughness. It is well known that the transverse rupture strength and fracture toughness of cermets are strongly dependent on the interface characteristics between the metal matrix and ceramic particles [18]. Toughening via crack deflection can be established for brittle materials [19], and the strain energy release rate of cermets, G_cermet_, can be estimated through the brittle fracture of Ti(C,N)/(Ti, W, Ta, Mo)(C,N) core/rim structure and the plastic rupture of (Co,Ni). The combined effects of the core/rim structure and Co/Ni binder can be described by:

G_cermet_ = (1 − V_binder_)G_core-rim_ + V_binder_σ_o_d_binder_χ (1)

where G_core-rim_ is the strain energy release rate of the core-rim structure, σ_o_ is the bulk flow stress of the binder, and χ is the function of bond strength in the rim/binder interface. χ can increase with increasing the bond strength of the interface. The drastically reduced lattice misfit at the core/rim/binder phase interfaces can stabilize the coherent interface by lowering the elastic strain energy [20] and improve the bond strength of the grain boundary, which can enhance the mechanical properties and abrasion resistance. Therefore, Ti(C,N)-based cermets prepared by the extra solid phase reaction can possess a core/rim/binder grain boundary with coherent structure, which can enhance the mechanical properties and abrasion resistance of the Ti(C,N)-based cermets.

## 4. Conclusions

Ti(C,N)-based cermets with ultrahigh transverse rupture strength can be prepared by the solid phase reaction and liquid phase sinter-HIP technique. It was found that the core/rim/binder grains can have a coherent interface structure at the grain boundary, which can be favorable to enhance the mechanical properties and improve the abrasion resistance of Ti(C,N)-based cermets. The successful synthesis of the coherent phase boundary should be suitable for the manufacture of high-quality Ti(C,N)-based cermets, and provide a novel approach to achieve excellent properties in cermets.

## Figures and Tables

**Table 1:** Properties of Ti(C,N) cermets prepared by solid phase reaction and sinter-HIP.

Specimen	Sintering Method	Density (g/cm^3^)	Relative Density (%)	Rockwell A Hardness	Vickers Hardness (GPa)	Fracture Toughness (MPam^1/2^)	Transverse Rupture Strength (MPa)
Present work	solid phase reaction + liquid phase sinter-HIP	7.120	99.8	92.3	1680 ± 20	11.6 ± 0.3	2810 ± 80
Present work	liquid phase sinter-HIP	7.105	99.6	92.2	1610 ± 10	9.6 ± 0.2	1820 ± 70
[10]	vacuum sintering	--	--	92.2	--	9.2	1804
[15]	vacuum sintering	--	--	90.3	1380	10.2	1505
[16]	hot pressing	--	--	91.0	1500	8.3	1200

## Supplementary Materials

The following are available online at www.mdpi.com/1996-1944/10/9/1090/s1, Figure S1: The curve of transverse rupture strength for cermets prepared by the extra solid phase, Figure S2: SEM micrographs of microstructure in Ti(C,N)-based cermets sintered at 1300 °C for 0 h (a) and 2 h (b), Figure S3: XRD patterns of Ti(C,N)-based cermets sintered at 1300 °C for 0 h and 2 h.
