# Supplementary material for: Enhanced Interface Structure and Properties of Titanium Carbonitride-Based Cermets with the Extra Solid Phase Reaction
Source: Materials (Basel). 2017 Sep 15;10(9):1090. doi: 10.3390/ma10091090 (PMC5615744; doi:10.3390/ma10091090)
Supplement: Supplementary file 1 [file materials-10-01090-s001.pdf]

materials

Communication

Enhanced Interface Structure and Properties of Titanium Carbonitride-Based Cermets with the Extra Solid Phase Reaction

Supplementary Materials

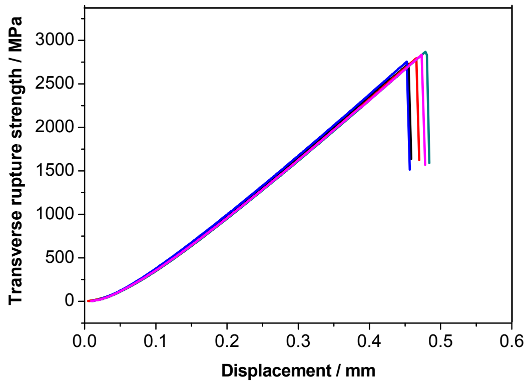

Figure S1. The curve of transverse rupture strength for cermets prepared by the extra solid phase reaction.

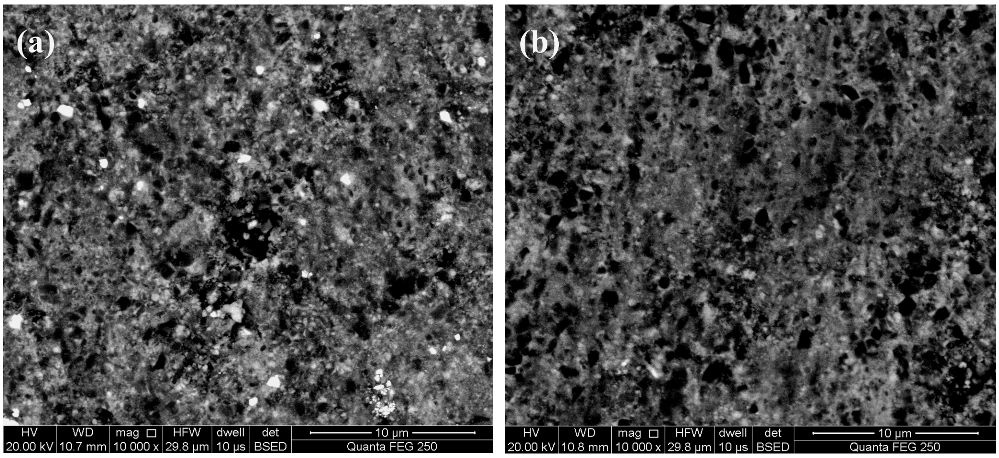

Figure S2. SEM micrographs of microstructure in Ti(C,N)-based cermets sintered at 1300 °C for 0 h (**a**) and 2 h (**b**).

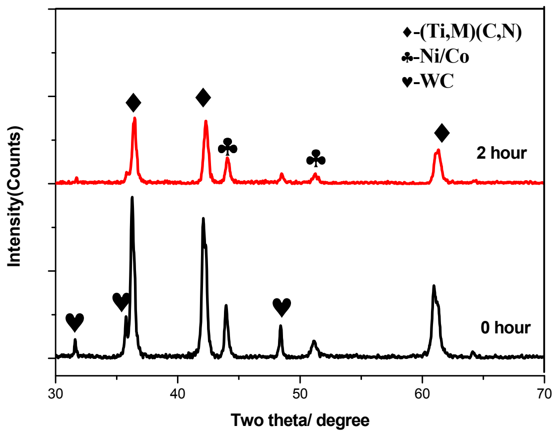

Figure S3. XRD patterns of Ti(C,N)-based cermets sintered at 1300 °C for 0 h and 2 h.
